# Prognosis of Patients with Papillary Thyroid Carcinoma Located in One Lobe Showing Lateral Node Metastasis in the Contralateral but Not Ipsilateral Compartment

**DOI:** 10.1155/2012/953603

**Published:** 2011-07-28

**Authors:** Yasuhiro Ito, Takuya Higashiyama, Yuuki Takamura, Kaoru Kobayashi, Akihiro Miya, Akira Miyauchi

**Affiliations:** Department of Surgery, Kuma Hospital, 8-2-35 Shimoyamate-dori, Chuo-ku, Kobe 650-0011, Japan

## Abstract

Papillary thyroid carcinoma (PTC) frequently metastasizes to the lymph node in lateral compartment, which can often be detected on preoperative ultrasonography (N1b). However, PTC located in one lobe showing contralateral but not ipsilateral N1b is not common. We analyzed the clinicopathological features and prognosis of 13 patients with PTC limited in one lobe showing contralateral but not ipsilateral N1b. Sizes of the primary lesions ranged from 0.8 cm to 3.0 cm and only 2 tumors showed extrathyroid extension. Metastatic lateral node measured from 0.6 to 3.1 cm. Ten patients showed pathological central node metastasis and 5 had minute PTC lesions in the contralateral lobe. However, 3 patients did not show either of these. None of the patients have developed carcinoma recurrence or died of carcinoma to date. Taken together, PTC located in one lobe with contralateral but not ipsilateral N1b is rare and generally shows an indolent behavior. Although most patients had central node metastasis and/or minute PTC lesions in the contralateral lobe, it is also possible for carcinoma cells to metastasize directly from primary lesions to the contralateral lateral node. Total thyroidectomy with central node dissection and therapeutic MND of the contralateral compartment may be an acceptable surgical design and bilateral MND might not be mandatory.

## 1. Introduction

Papillary thyroid carcinoma (PTC) generally shows an indolent character. However, it is also well known that PTC frequently metastasizes or recurs to regional lymph node. There are two compartments vulnerable to PTC, central and lateral compartments. The central compartment was located adjacent to the thyroid and can be dissected through the same wound as that for thyroidectomy. Therefore, routine central node dissection for PTC has been performed in many institutions, including ours, in Japan. It is also recommended in the Japanese guidelines [1, 2], although not in various Western guidelines [3–6]. Dissection of the lateral compartment (modified radical neck dissection (MND)) is mandatory for PTC with clinical lateral node metastasis (N1b) detected on palpation or on imaging studies (therapeutic MND). However, the indication for prophylactic MND for PTC patients who are not classified as N1b remains debatable. 

 We previously demonstrated that N1b was a predictor of poor prognosis in PTC patients [7]. Especially, N1b measuring 3 cm or larger or having extranodal tumor extension are signs of high-risk (poor disease-free survival (DFS) and cause-specific survival [CSS]), while other N1b patients were classified as intermediate risk (poorDFS but not CSS) [8, 9]. For PTC located in one lobe, lateral node metastasis firstly occurs ipsilateral to the primary lesion, although bilateral lateral node metastasis can be detected in advanced cases. However, surgeons can encounter PTC located in one lobe with contralateral but not ipsilateral N1b on preoperative imaging studies, and the appropriate extent of lymph node dissection in such cases remains uncertain. In this study, we investigated the details of 13 patients with PTC located in one lobe with contralateral but not ipsilateral N1b in order to elucidate its biological characteristics and behavior of such lesions.

## 2. Patients and Methods

We reviewed the records of patients who underwent initial surgery for PTC between 1995 and 2011 and found 13 patients with PTC located in one lobe while lateral node metastases contralateral, but not ipsilateral, to primary lesions were detected on preoperative imaging studies. We excluded patients who had other thyroid malignancies such as follicular carcinoma, anaplastic carcinoma, medullary carcinoma, and thyroid malignant lymphoma. Patients having nodules suspected of malignancy in the contralateral lobe were also excluded. Primary lesions of these patients were diagnosed as PTC on fine needle aspiration biopsy (FNAB). Lateral node metastasis was preoperatively diagnosed on ultrasonographic findings and, although not in all cases, FNAB for nodes with thyroglobulin measurement of wash-out of needles [10]. On pathological examination, these patients were diagnosed as having PTC with metastasis. After surgery, two patients underwent whole body scan using 13 mCi of radioactive iodine (RAI), but no abnormal uptake was detected. All patients were followed postoperatively by ultrasonography and chest CT or roentgenography at least once per year.

## 3. Results

Table 1 summarizes the background data and findings on preoperative imaging studies of 13 patients with PTC located in one lobe and contralateral but not ipsilateral N1b. Seven of 13 patients (53%) were males and patients ages ranged from 24 to 71 years (average 52 years). Three patients (23%) had multiplicity of PTC, although all tumors were limited to one lobe. Four patients (31%) showed nodules in the contralateral lobe, which were diagnosed as benign nodules on ultrasonography or cytology. Sizes of the contralateral metastatic nodes ranged from 0.6 to 3.1 cm and only 1 patient (7%) had metastasis larger than 3 cm. Central node metastasis was preoperatively detected in 5 patients (38%) in imaging studies. 

 All patients underwent total thyroidectomy and entire central node dissection (Table 2). Five patients (38%) underwent unilateral (contralateral to primary lesions) and the remaining 8 (62%) underwent bilateral MND. One patient (number 12) showed extension of primary lesion to the recurrent laryngeal nerve, which was dissected and reconstructed. Metastatic lateral node invaded to the phrenic nerve in case number 6. In 3 patients (numbers 6, 11, and 13), findings in the central compartment were negative. Seven of 8 patients who underwent bilateral MND were positive for metastasis to the ipsilateral lateral compartment. Although there were no carcinoma lesions in the contralateral lobe on preoperative ultrasonography, 5 patients (38%) had minute PTC lesions in the contralateral lobe on pathological examination. Three patients (numbers 6, 11, and 13) were negative for central node metastasis and did not have PTC in the contralateral lobe. 

The postoperative follow-up period ranged from 1 to 154 months (average 69 months). None of these patients have shown carcinoma recurrence or died of carcinoma to date. Although none of these patients underwent RAI ablation, thyroglobulin levels of all patients except number 13, who only recently underwent surgery, were kept at undetectable levels under TSH suppression and have not shown elevation during followup.

## 4. Discussion

Of the two compartments of regional lymph nodes, the central compartment, is more adjacent to the thyroid than the lateral compartment. Therefore, it is apt to be considered that lateral node metastasis occurs via central node metastasis. However, we have previously shown that the incidence of central-positive and lateral-negative patients did not much differ from that of central-negative and lateral-positive patients, who underwent central node dissection and MND, indicating that lateral node metastasis can occur directly from the thyroid [6, 11]. Generally, however, lateral node metastasis occurs ipsilateral to the primary lesion, although advanced cases can show lateral node metastasis bilaterally. 

 In this study, we investigated rare PTC cases having primary lesions in one lobe with contralateral but not ipsilateral N1b. In this series, 2 patients showed extrathyroid extension (numbers 3 and 12), but case number 3 extended only to the recurrent laryngeal nerve and number 12 extended only to the sternohyoid muscle. Furthermore, only one patient (number 12) showed node metastasis 3 cm or larger. Therefore, the number of high-risk patients included in our series was limited and most cases showed a mild and indolent character. 

On pathological examination, 10 patients and 5 patients had central node metastasis and minute PTC lesions in the contralateral lobe, respectively. It is therefore suggested that PTC of these patients metastasized to the lateral compartment of the contralateral side from central compartment or from small PTC lesions in the contralateral lobe. However, 3 patients did not show central node metastasis or minute PTC lesions in the contralateral side, indicating that PTC can metastasize directly to the contralateral lateral compartment and that there are various pathways for metastasis from primary lesions to regional lymph nodes. 

 Extent of lymph node dissection for patients with only contralateral N1b seems controversial. Seven of 8 patients who underwent bilateral MND were positive for metastasis in the ipsilaterallateral compartment, although there was no metastasis detected on preoperative imaging studies. Indeed, ultrasonography is the most useful tool to preoperatively evaluate node metastasis, but the negative predictive value of lateral node metastasis was as low as 43% in our previous study of over 3000 patients, indicating that more than half of the patients with PTC had latent lateral node metastasis undetectable on ultrasonography [6]. The incidence of latent lateral node metastasis increases with tumor size, and around 90% of patients with PTC larger than 4 cm had latent ipsilateral lateral node metastasis, while the incidence of microcarcinoma was around 40% [12]. However, such a latent metastasis is not immediately life threatening and second surgery after the metastasis becomes overt is generally adequate, which is the reason that prophylactic MND is not recommended in various guidelines [2–5]. In our series, none of 5 patients who underwent unilateral MND showed recurrence to lateral lymph nodes ipsilateral to the primary lesionsduring followup ranging from 26 to 142 months. It is therefore suggested that prophylactic MND ipsilateral to the primary lesion is not mandatory for such patients. Decision whether bilateral MND is performed for individual patient should depend on other patients' characteristics such as patient age, gender, tumor size, and extrathyroid extension.

 In summary, PTC located in one lobe with contralateral but not ipsilateral N1b is rare and generally, such cases show a comparably mild behavior. Although most patients had central node metastasis and/or minute PTC lesions in the contralateral lobe, it is possible for carcinoma cells to metastasize directly from primary lesions to the contralateral lateral node. Total thyroidectomy with central node dissection and therapeutic MND of contralateral side is an acceptable surgical design and bilateral MND may not be mandatory.

## Figures and Tables

**Table 1:** Backgrounds and preoperative evaluation of 13 patients with contralateral but not ipsilateral N1b.

Case number	Gender	Age	Tumor size (cm)	Tumor multicentricity	Benign nodules at contralateral side	Central node metastasis	N1b size (cm)
1	Female	26	3.0	No	No	No	0.7
2	Female	54	1.1	No	Yes	No	0.8
3	Male	55	3.3	No	No	Yes	1.5
4	Male	24	4.5	No	No	Yes	0.9
5	Female	46	1.6	Yes	No	Yes	1.9
6	Female	69	0.7	No	No	No	1.6
7	Male	53	1.4	Yes	No	No	2.2
8	Male	57	1.9	No	No	No	0.6
9	Male	45	2.0	No	No	No	1.0
10	Male	50	3.0	No	Yes	Yes	0.8
11	Female	60	0.8	Yes	Yes	No	1.6
12	Female	67	1.9	Yes	Yes	Yes	3.1
13	Male	71	0.9	No	No	No	1.9

**Table 2:** Intraoperative and pathological findings of 13 patients with contralateral but not ipsilateral N1b.

Case number	Lymph node dissection	Extrathyroid extension	Extranodal extension	Pathological central metastasis	Pathological ipsilateral metastasis	Presence of carcino a in the contralateral lobe	Follow-up period (mos)
1	Bilateral	No	No	Yes	Yes	Yes	154
2	Unilateral	No	No	Yes	?	No	142
3	Bilateral	Yes	No	Yes	Yes	No	130
4	Bilateral	No	No	Yes	Yes	Yes	116
5	Unilateral	No	No	Yes	?	Yes	89
6	Unilateral	No	Yes	No	?	No	56
7	Bilateral	No	No	Yes	Yes	Yes	47
8	Bilateral	No	No	Yes	no	no	44
9	Bilateral	No	No	Yes	Yes	No	42
10	Bilateral	No	No	Yes	Yes	Yes	37
11	Unilateral	No	No	No	?	No	26
12	Bilateral	Yes	No	Yes	Yes	No	17
13	Unilateral	No	No	No	?	No	1
